# Large-sized Fetal Striatal Grafts in Huntington’s Disease Do Stop Growing: Long-term Monitoring in the Florence Experience

**DOI:** 10.1371/currents.hd.c0ad575f12106c38f9f5717a8a7d05ae

**Published:** 2014-08-04

**Authors:** Mario Mascalchi, Stefano Diciotti, Marco Paganini, Andrea Bianchi, Andrea Ginestroni, Letizia Lombardini, Berardino Porfirio, Renato Conti, Nicola Di Lorenzo, Gabriella Barbara Vannelli, Pasquale Gallina

**Affiliations:** Quantitative and Functional Neuroradiology Research Unit, Meyer Children and Careggi Hospitals, Florence, Italy; Department of Clinical and Experimental Biomedical Sciences, University of Florence, Florence, Italy; Department of Electrical, Electronic, and Information Engineering “Guglielmo Marconi”, University of Bologna, Cesena, Italy; Neurology Unit, Careggi Hospital, Florence, Italy; Neuroradiology Unit, Careggi Hospital, Florence, Italy; Hematology Unit, Careggi Hospital, Florence, Italy; Department of Clinical and Experimental Biomedical Sciences, University of Florence, Florence, Italy; University of Florence; Department of Surgery and Translational Medicine, University of Florence, Florence, Italy; Department of Clinical and Experimental Medicine, University of Florence, Florence, Italy; Department of Surgery and Translational Medicine, University of Florence, Italy

## Abstract

Development of six large nodules of solid tissue after bilateral human fetal striatal transplantation in four Huntington’s disease patients has raised concern about the safety of this experimental therapy in our setting. We investigated by serial MRI-based volumetric analysis the growth behaviour of such grafts. After 33-73 months from transplantation the size of five grafts was stable and one graft showed a mild decrease in size. Signs neither of intracranial hypertension nor of adjuctive focal neurological deficit have ever been observed. This supports long-term safety of the grafting procedure at our Institution.

## Introduction

Huntington’s disease is an incurable neurodegenerative disorder for which human fetal striatal transplantation is being explored as an experimental approach with uncertain long-term results.^1^^,^ 2 ^,^ 3 Recently, Paganini et al.^4^ reported that fetal striatal grafting slowed the progression of motor and cognitive decline in patients participating in the Florence transplantation program. This renewed the interest in such a therapeutic strategy in Huntington’s disease.^5^ This notwithstanding, several challenges remain to be faced, not least the long-term fate of the graft. In most Huntington’s disease transplantation studies the grafted material showed some form of development, but the way and the extent of the growth greatly varied both among and within trials.^6^ In four patients of the Florentine series, six of eight grafts resulted in the growth of large tissue nodules overcoming the size of native striatum.^7^ This behaviour was deemed as “overgrowth” and raised much concern about the safety of our transplantation procedure.^8^ In particular, it was anticipated a possible adverse effect of the “marked cellular overgrowth” on the clinical status of the patients with particular emphasis on the risk of development of fatal mass lesions and even the ultimate necessity of surgical removal of graft tissue.^8^ More recently, Cisbani and Cicchetti^6^ re-launched worries on the outcome of our large-sized grafts. We report here the demonstration by magnetic resonance imaging volumetric analysis that the growth of these grafts stopped within the fourth quarter after transplantation and remained stable in the long term.

## Methods

Ethics Statement. The present study has been conducted according to the Declaration of Helsinki principles. The patients provided written informed consent. The transplantation program was authorized by the Italian National Health Institute, National Transplantation Centre (upon approval by Health Ministry, Consiglio Superiore di Sanità, Sessione XLV, Sezione II, 7/21 and 9/22/2005, and acceptance by the National Bioethics Committee).

Human fetal striatal transplantation at our Institution implies bilateral (two months apart) caudate-putaminal stereotactic-robotic (NeuroMate Robotics System, Schaerermayfield, France) grafting of a tissue suspension obtained from both whole ganglionic eminencies of a single legally aborted human fetus (9–12 weeks gestation).^7^ All magnetic resonance imaging examinations of these four patients (n=20, 3–7 each) from baseline (9–12 months after grafting, Figure 1a) to those obtained at the last available follow-up (range 33-73 months after grafting) were considered here. Examinations were carried out under mild sedation on the same 1.5T unit and the protocol included T2-weighted and contrast-enhanced high-resolution 3D T1-weighted images. Using the Striker navigation system workstation (Howmedica Leibinger, Freiburg, Germany) a neuroradiologist manually contoured the entire grafts in axial 3D T1-weighted MPRAGE (1 mm thick, 256x256 matrix) images with real time control of the segmentation on images reformatted on the sagittal and coronal planes available on complementary viewing windows. The volume of the grafts was automatically calculated. At least two measurements per graft per scan (total 81, mean 2.9) were taken at a 2-week interval by the same operator.

## Results

The coefficient of variations (CV = standard deviation/mean) were all below 10%. The largest graft was by far n. 4 with a mean volume of 42.0±1.8 mL and 43.0±1.9 mL at 10 and 18 months after surgery, respectively, then decreased by 20% and remained unchanged at 45-54 months. The volumes of the other five grafts at baseline ranged between 5.3 and 15.9 mL. Their size remained stable, namely the regression lines of volumes over time had slopes not significantly different from 0. Mean volume measurements over time are displayed in Figure 1b.

**Figure:**
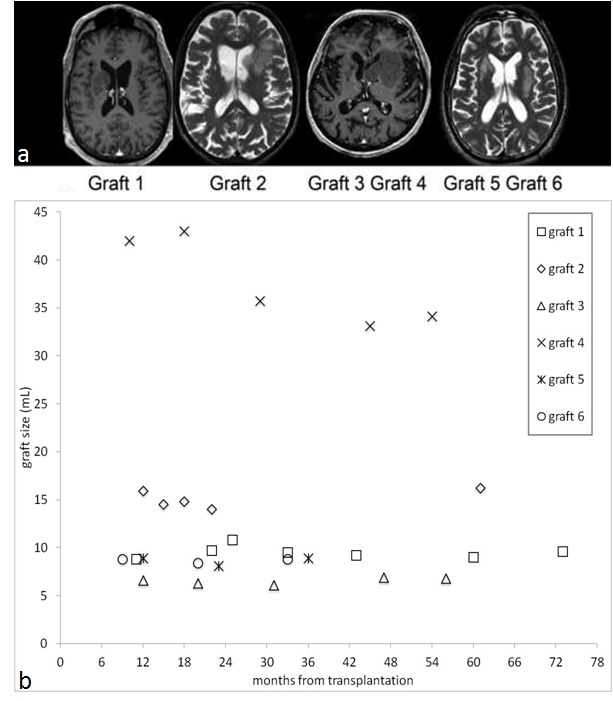
Figure 1: (a) Contrast-enhanced T1-weighted (grafts n. 1, 3 and 4) and T2-weighted (grafts n. 2, 5 and 6) magnetic resonance images obtained 9-12 months after human fetal striatal transplantation in four Huntington’s disease patients in which grafts attained a large size. (b) Mean of repeated volume measurements for each of the six grafts at each patient-specific magnetic resonance examination follow-up time are plotted. A series of linear mixed models with graft-specific random intercepts were fitted. Linearity tests satisfied the simple regression model for grafts n. 1, 2, 3, 5, and 6. The estimated slopes were, respectively, -0.004, 0.026, 0.010, 0.002, and 0.001, all not significantly different from 0. In the case of graft n. 4, the appropriateness of linear regression model was instead rejected.

## Discussion

The risk of uncontrolled proliferation is intrinsic to cell-based therapy.^9^ However, to the best of our knowledge, death attributed to fetal graft overgrowth occurred only in two patients with Parkinson’s disease.^10^^,^ 11 In one patient the striatal and intraventricular grafts were contaminated with tissue from multiple germ layers and the solid intraventricular tissue led to ventricular obstruction.^10^ In the other patient the midbrain graft was contaminated with choroid plexuses and death was due to mass effect created by a cystic lesion.^11^ In one patient with Huntington’s disease, who received bilateral intrastriatal transplants of lateral ganglionic eminence from several fetuses, multiple solid and one ependymal cystic mass lesions were discovered five years after transplantation, but both remained stable for four years until death which followed complications of disease progression.^12^

In the four patients reported here, a less pronounced motor and cognitive decline without any symptom or sign of mass effect was demonstrated.^4^ The results of the present investigation indicate that the large size attained by grafts in these patients is not followed by and does not imply progressive uncontrolled growth. This is in line with [^18^F]fluorodeoxyglucose positron emission tomography data in the same patients, showing striatal and cerebral cortical metabolic increase compared to the pre-surgical evaluation two years after transplantation.^4^^,^ 7 The slight relative metabolic decrease observed at later follow-up^4^ would be inconsistent with a neoplastic drift of the grafts. The biological mechanisms regulating the development of fetal striatal tissue into a diseased brain deserve as much understanding as the clinical impact of this growth on the transplanted patients. Sarchielli et al.^13^ extensively characterized primary cell cultures from human fetal striatal primordium. These cells express neurotrophins able of maintaining cell plasticity, as well as promoting neurogenesis, migration and survival.^13^ Moreover, Gallina et al.^14^ recently showed that also in a graft of gestational age comparable with those of fetuses used as source in the present cases, cells exhibit a surface biomarker code, namely CD15, CD24, and CD29, which regulates neural cell proliferation and differentiation.^15^ Overall, available evidence is consistent with the view that the large size attained by some grafts in Huntington’s disease transplantation is the result of a time-scheduled and self-limiting development of the striatal primordium.^14^ We are conscious that present knowledge is not sufficient to ensure that these grafts will maintain their quiescent status and, therefore, further surveillance is warranted.

## Competing Interests

The authors have declared that no competing interests exist.
